# The Physicochemical Synergy Effect of Nanosecond Pulsed Electric Fields (nsPEF) and cisplatin on reversing chemoresistance of bladder cancer

**DOI:** 10.1371/journal.pone.0346699

**Published:** 2026-04-16

**Authors:** Danjing Guo, Xiao Li, Xiaobo Xu, Xinhua Chen, Hongqiang Cheng, Junhui Jiang

**Affiliations:** 1 Division of Hepatobiliary and Pancreatic Surgery, Department of Surgery, The First Affiliated Hospital, Zhejiang University School of Medicine, Hangzhou, Zhejiang, China; 2 NHC Key Laboratory of Combined Multi-organ Transplantation, Key Laboratory of Organ Transplantation, Hangzhou, Zhejiang, China; 3 Zhejiang University School of Medicine, Hangzhou, Zhejiang, China; 4 Department of Urology The First Aﬃliated Hospital of Ningbo University, Ningbo, Zhejiang, China; National Research Council: Consiglio Nazionale delle Ricerche, ITALY

## Abstract

Nanosecond Pulsed Electric Field (nsPEF) is an intense electrical pulse technology with ultra-short duration (nanoseconds), capable of generating electric field intensities up to several kV/cm. By inducing irreversible electroporation for tumor tissue ablation, nsPEF triggers apoptosis in cancer cells, stimulates anti-tumor immune responses, and enhances exogenous gene transfection. As a primarily non-thermal technique, it offers advantages such as minimal tissue damage, high targeting efficiency, short treatment duration, and compatibility with other therapies. Currently demonstrating broad application potential in cancer treatment. Urothelial Bladder Carcinoma (UBC), accounting for over 90% of bladder cancers, originates from the transitional epithelium of the bladder’s urothelium and represents the most common malignant tumor in the urinary system. Cisplatin (CDDP), a key chemotherapeutic agent for UBC, is particularly effective for recurrent or advanced-stage patients. However, repeated use often leads to drug resistance. This study investigates the cytotoxic effects of nsPEF on UBC cells and its inhibitory mechanisms against drug-resistant UBC. Through sustained gradient concentration induction of CDDP, we successfully established drug-resistant cell lines T24/CDDP and TCC/CDDP. After treatment with nsPEF on parental cell lines (T24, TCC) and their drug-resistant variants (T24/CDDP, TCC/CDDP), all cells exhibited time-and dose-dependent sensitivity. Western blot analysis revealed that the basal expression of γ-H2AX was significantly reduced in the drug-resistant cells (T24/CDDP, TCC/CDDP), while nsPEF treatment significantly upregulated its expression. In vivo experiments further demonstrated that nsPEF exhibited significant antitumor efficacy against tumor-bearing mice from T24/CDDP. Overall, nsPEF effectively induces apoptosis in T24, TCC, and their drug-resistant variants, with particularly potent effects on drug-resistant UBC cells. This enhanced effect may be attributed to nsPEF-induced more severe DNA damage in drug-resistant cells, as manifested by elevated γ-H2AX expression.. This study provides experimental evidence for applying nsPEF to overcome chemotherapy resistance in UBC.

## Introduction

Bladder cancer is one of the ten most common cancers worldwide. According to the Global Cancer Observatory GLOBOCAN statistics for 2020 [1], there were approximately 573,000 new cases of bladder cancer and 213,000 deaths. Urothelial carcinoma is the most common type of bladder cancer, accounting for over 90% of all bladder cancer cases [2]. Current treatments for bladder cancer mainly include surgical resection, immunotherapy, radiation therapy, and chemotherapy. However, each treatment has its unique advantages and is accompanied by certain side effects. Surgical resection is the primary treatment, but many patients are not candidates for radical resection [3]. Immunotherapy activates the patient’s immune system to recognize and eliminate cancer cells. However, immunotherapy for bladder cancer lacks reliable predictive biomarkers [4]. Radiation therapy is used when postoperative tumor residue causes extravesical invasion in bladder cancer, but it cannot be used for patients with severe renal failure [5]. Once bladder cancer progresses, patients usually need to undergo radical cystectomy combined with chemotherapy, such as cisplatin [6,7]. However, chemotherapy has certain toxic side effects, mainly affecting gastrointestinal and hematopoietic function, and patients may experience symptoms such as nausea and vomiting, hair loss, and decreased white blood cells and platelets. The underlying mechanisms of chemoresistance remain unclear.

The recurrence is relatively high, with about 10%-15% of patients’ tumors developing into malignant muscle-invasive bladder cancer, and the 5-year survival rate drops to 5% [8]. Combination chemotherapy based on cisplatin has been widely used in the treatment of muscle-invasive bladder cancer and has improved survival rates [9]. The effectiveness of cisplatin-based chemotherapy remains at 50% due to its high tendency to develop resistance [10]. Cisplatin resistance is caused by decreased intracellular platinum accumulation [11] or increased inactivation [12,13], enhanced DNA repair capacity [14], increased inhibition of cell apoptosis, and stimulation of survival signal transduction [15]. The novel strategy to reverse cisplatin resistance is in urgent need.

Nanosecond pulsed electric field (nsPEF) is an emerging novel pulsed electric field technology and has gradually become a research hotspot [16,17]. Reports have shown that nsPEFs are effective in treating many tumors, such as melanoma or prostate cancer [16,18]. nsPEFs can produce various effects depending on the parameters applied (voltage, pulse duration, pulse output frequency, and number of pulses). When the voltage exceeds the electroporation threshold, reversible pores are formed on the cell membrane [19], thereby increasing the delivery of chemotherapeutic drugs to tumor cells [20]. When the voltage greatly exceeds the electroporation threshold, it leads to irreversible electroporation (IRE) [20,21], during which the cell membrane permanently loses its integrity, cytoplasmic contents leak, and cell apoptosis occurs. Changes in pulse duration also lead to different responses in tumor cells. Microsecond pulses usually directly cause cell membrane permeabilization, while nsPEF can penetrate the cell membrane and directly target organelles such as the mitochondria, nucleus, and endoplasmic reticulum, inducing cytoskeletal disruption and subsequent cell damage [22,23]. High-frequency output pulses are perceived by cells as a single cumulative pulse [24], while low-frequency pulses are perceived as a series of pulses [23]. In addition, nsPEF modulates the tumor microenvironment, alters the profiles of immune cells and cytokines, inhibits tumor angiogenesis, and prevents tumor metastasis and recurrence [25]. It is known that nsPEFs have a toxicity effect on sensitive bladder cancer [26], but there are few reports on the efficacy of nsPEF for cisplatin-resistant bladder cancer and the underlying molecular mechanisms. Therefore, this study induced and established two bladder cancer resistant cell lines, T24 CDDP and TCC CDDP, to investigate the efficacy of nsPEFs combined with cisplatin and its role and mechanism in improving bladder cancer resistance.

The innovative combination of nanosecond pulse electric field (nsPEF) and low-dose cisplatin (CDDP) demonstrates significant synergistic cytotoxic effects against multidrug-resistant bladder cancer (UBC) cells. This integrated strategy enhances localized ablation therapy through nsPEF, improves drug delivery efficiency, boosts chemotherapy penetration, and reverses tumor cell resistance, offering a novel approach for patients intolerant to CDDP or resistant to conventional treatment.

## 2 Materials and methods

### 2.1 Experimental cells

Human bladder cancer transitional epithelial tumor cell line (T24, RRID: CVCL_0554) and human bladder cancer transitional epithelial tumor cell line (TCC, RRID: CVCL_1738) were purchased from the US ATCC Cell Bank (Cell Resource Center of Shanghai Institutes of Biological Sciences, Chinese Academy of Sciences, Shanghai, China).

### 2.2 Experimental animals

Immunodeficient mice BALB/ c nude mice (all 4 weeks old, male, weight 20-22g) were purchased from Hangzhou Ziyuan Experimental Animal Co., Ltd. and raised in the SPF animal breeding room of an affiliated hospital of Zhejiang University Medical College. Animal experiments were approved by the Laboratory Animal Ethics Committee of the First Affiliated Hospital of Zhejiang University Medical School. Animals were euthanized by carbon dioxide asphyxiation. The isoflurane inhalation was used for anesthesia induction and buprenorphine for postoperative pain management. Efforts made to alleviate suffering through daily health monitoring, provision of soft bedding. Procedures involving experimentation on animal subjects are done in accord with the National Research Council’s guide for the care and use of laboratory animals.

### Ethical considerations, compliance and protocol approval

This study is compliant with guidelines on animal experimentation of the Institutional Animal Care and Use Committee of Zhejiang University. Animal experiments were approved and supervised by the Laboratory Animal Ethics Committee of the First Affiliated Hospital of Zhejiang University Medical School (IACUC#2025−074). Procedures involving experimentation on animal subjects are done in accord with the National Research Council’s guide for the care and use of laboratory animals.

### 2.3 Cisplatin configuration

Cisplatin (MedChemExpress LLC, Shanghai, China. LOT No. HY-17394 CAS No. 15663-27-1), is diluted as 1 mg/mL storage liquid. On use add it to medium to the specified concentration.

### 2.4 Chemoresistant cell lines

When the cell density reaches 80% (logarithmic growth phase) and the cells grow stably at this concentration, T24/TCC cells and the half-maximal inhibitory concentration (IC50) of cisplatin in each parental cell line was determined by CCK 8 method IC50. The medium was then replaced with a medium containing 0.1 μg/mL of cisplatin, and the medium containing Cisplatin was removed after 24 h, and replaced with fresh normal medium for continued culture. When the cell density reaches 80%, the cells will grow to the logarithmic phase until the cells can grow steadily at this concentration. After increasing the concentration of cisplatin to continuously induce cells until the cells can grow stably in complete medium containing 2.5 μg/L cisplatin, the IC50 of each resistant cell line was determined by CCK 8, and the chemoresistance index was calculated according to the IC50, chemoresistance index (RI) = IC50 (resistant cells)/ IC50 (parental cells). A cell line was defined as chemoresistant when the RI was greater than 3

### 2.5 CCK-8 assay

Take the cells in the logarithmic growth phase, wash them twice with 1 PBS, then digest the cells with trypsin. Cells were seeded into 96-well plates at a density of 5 × 10³ cells per well in 100 μL of complete medium together with five replicate wells, alongside control wells containing cells without cisplatin (negative control) and medium only (blank control). The plates were then incubated under standard culture conditions (37°C, 5% CO₂) for 48 hours. Following incubation, 10 μL of CCK-8 solution (Biyuntian, Shanghai, China) was added to each well. The absorbance (OD value) of each well was measured at a wavelength of 450 nm and cell viability rate was calculated.

### 2.6 nsPEFs ablation on chemoresistant cells

A nanosecond pulsed electric field system (Pulsed Electric Field in Medical Application Lab, Hangzhou, China) delivered an ultrashort pulse of 30 kV high electric field with a duration of 300 nanoseconds. Pulse width: 300 ns, field strength: 30 kV/cm, maximum pulse energy ≤1.2 J, frequency: 1 Hz, number of pulses was adjustable and set according to the experiment. Electroporation Cuvettes (BioRad, USA, 0.4 cm gap) were sterile electroporation cuvettes as the cell container to be treated by nsPEFs.

T24/ T24 CDDP and TCC/ TCC CDDP cells in the logarithmic growth phase were washed twice with 1 PBS, and then digested with trypsin. Cell digestion was observed under a microscope, trypsin was discarded, serum-containing media was added to terminate the digestion, and cell suspension was made. The cells were counted as 5 × 103 cells/ well, and 3 additional wells in each group to calculate the required cell amount. After group treatment, 100 μl of diluted cell suspension in each well was seeded in a 96-well plate. The groups included blank control group (no cells), cell control group (no nsPEFs) and nsPEFs experimental group (250 pulses, 500 pulses, 750 pulses, 1000 pulses), placed in a 37℃, 5% CO2 (CO2) incubator for 24 h, 48 h and 72 h. After the corresponding time, the medium was removed, 100 μ l of complete medium containing 10% CCK 8 reagent was added to each well and incubated in 37℃ of 5% carbon dioxide (CO2) for 90 min. The absorbance (OD) at 450 nm. Meanwhile, the half inhibitory concentration of nsPEFs (IC50) was calculated using GraphPad Prism 8.0.

### 2.7 Cellular flow experiments

The T24/ T24 CDDP and TCC/ TCC CDDP cells in the logarithmic growth phase were prepared into a cell suspension and counted. Cells were counted as 5 × 104 cells/ well, and 2 additional wells for each group to calculate the number of cells needed, and group-treated cells were seeded into 6-well plates. The groups included cell control group (without chemo/ nsPEFs) and dosing group (0.3125 μg/L, 0.625 μg/L, 1.25 μg/L, 5 μg/L, 10 μg/L, 20 μg/L), and nsPEFs group (250 pulses, 500 pulses, 750 pulses, 1000 pulses) in 37℃, 5% CO2 (CO2) incubator for 24 h, 48 h and 72 h. After the corresponding treatment time, the original supernatant was transferred into a 15 L centrifuge tube, the cells were digested with trypsin, centrifuged at 1000 rpm for 5 min, the supernatant was washed twice with 4℃ pre-cooled 1 PBS, the supernatant was discarded, 100 μ l 1 Binding Buffer was resuspended in each tube, and 5 μl Annexin V and 5 μl PE dye were added and incubated in the light for 15 min. Supplement 400 μl 1 Binding Buffer per tube and test by machine. Flows the resulting data were analyzed using flowJo10.

### 2.8 Cell protein extraction, quantification and western blot analysis

The T24 CDDP and TCC CDDP cells in the logarithmic growth phase were prepared into cell suspension and counted. Cells were seeded into 6-well plates. The groups included the cell control and dosing (0.3 μg/L), nsPEFs (125 pulses) and nsPEFs cisplatin (0.3 μg/L + 125 pulses) and cultured in a 37℃, 5% carbon dioxide (CO2) incubator for 1 h. Standard protein (original concentration 2 μg/ μl) was serially diluted in a 96-well plate to a series of different concentrations (1 μg/ μl, 0.5 μg/ μl, 0.25 μg/ μl, 0.125 μg/ μl, 0.0625 μg/ μl, 0.03125 μg/ μl, 0.015625 μg/ μl, 0 μg/ μl). The liquid volume of each well was 20 μl and used to make the standard curve. Determine the absorbance value of the wavelength of 570 nm per well using a microplate reader, draw the standard curve, and calculate the protein concentration of proteins in each group of samples. Samples were diluted to 2 μg/ μl using protein lysate and 4 loading buffer liquid for protein immunoblot blot. Put the 4-20% 12-well precast glue into the electrophoresis tank, and add an appropriate amount of WB electrophoresis solution. PVDF membrane is wrapped with sponge clip and placed in the membrane transfer groove into the membrane transfer apparatus for membrane transfer. After turning the membrane, the PVDF membrane was placed in the blocking solution containing 5% non-fat milk powder and blocked for 1 h by shaking at room temperature. After sealing, the protein was rinsed twice with 1 TBST solution. The PVDF membrane was cut according to the protein molecular weight, and the band where the target protein was removed and incubated in the corresponding primary antibody for 4℃ overnight. The next day, the bands were rinsed with 1 T BST solution for 10 min 3 times, incubated in the secondary antibody of the corresponding species for 1 h under shaking, and rinsed with 1 T BST solution for 10 min. ECL chemiluminescent solution was added to completely cover the strip and develop color and photograph using Fluor ChemTM imaging system.

### 2.9 nsPEF ablation of the chemoresistant bladder cancer model in nude mice

T24 CDDP cells at a concentration of 2 × 107 cells/ L were seeded subcutaneously in the back of BALB/ c nude mice. After the tumor grew to about 0.8 × 00.8cm2 in size, the nude mice were randomly divided into 4 groups: control group, single cisplatin treatment group, single nsPEFs treatment group and combined treatment group, with 6 mice in each group. The control group was not treated; single cisplatin group (2.5 mg/ kg); single nsPEFs treatment group (500 pulses); and the combined treatment group (2.5 mg/ kg + 500 pulses). The nsPEF monotherapy group received ablation with two vertically placed needles (250 pulses each), 0.5 cm and 30kV/ cm electric field strength. The combined treatment group received the same intensity as the single nsPEFs treatment group. Observe the growth status of nude mice and tumor regularly, weigh the weight of nude mice every two days. In case of tumor or tumor rupture over 2.0 cm, animals were euthanized according to animal ethics and welfare regulations, nude mice were killed 14 days later, and heart, liver, spleen, lung, kidney and tumor tissues were collected for subsequent analysis. The tissues were stained for HE, immunohistochemical staining (IHC) and TUNEL.

### 2.10 The Combination Index (CI) for evaluating the synergistic effect

To assess the combined effect of these two “drugs”, the effect values of nsPEF alone under different pulse number conditions, the effect value of CDDP alone at a fixed concentration, and the effect value of their combination were measured separately. Cell viability was determined using the Cell Counting Kit-8 (CCK8) assay, which is a reliable method for quantifying viable cells based on the reduction of a water-soluble tetrazolium salt to a formazan dye by dehydrogenase enzymes in living cells. The absorbance of the formazan product was measured using a microplate reader at a wavelength of 450 nm, and cell viability was calculated relative to the control group (without nsPEF or CDDP treatment).Subsequently, the Combination Index (CI) was calculated using CompuSyn software, a specialized tool for quantitative analysis of drug-drug or drug-physical therapy combinations based on the Chou-Talalay method. This method integrates the dose-effect relationships of individual treatments and their combinations to determine the nature and degree of their interaction.

### 2.11 Statistical analysis

Experimental data are presented as the mean number ± SD. Differences between multiple groups were analyzed using Graphpad Prism 8 software, tested by One-way ANOVA test, and corrected using Turkey’s HSD. P < 0.05 was considered statistically different (* P < 0.05, * * P < 0.01, * * * P < 0.001, and * * * * P < 0.0001).

## 3 Result

### 3.1 Establishment and characterization of CDDP-resistant bladder cancer cell lines

**Fig 1:**
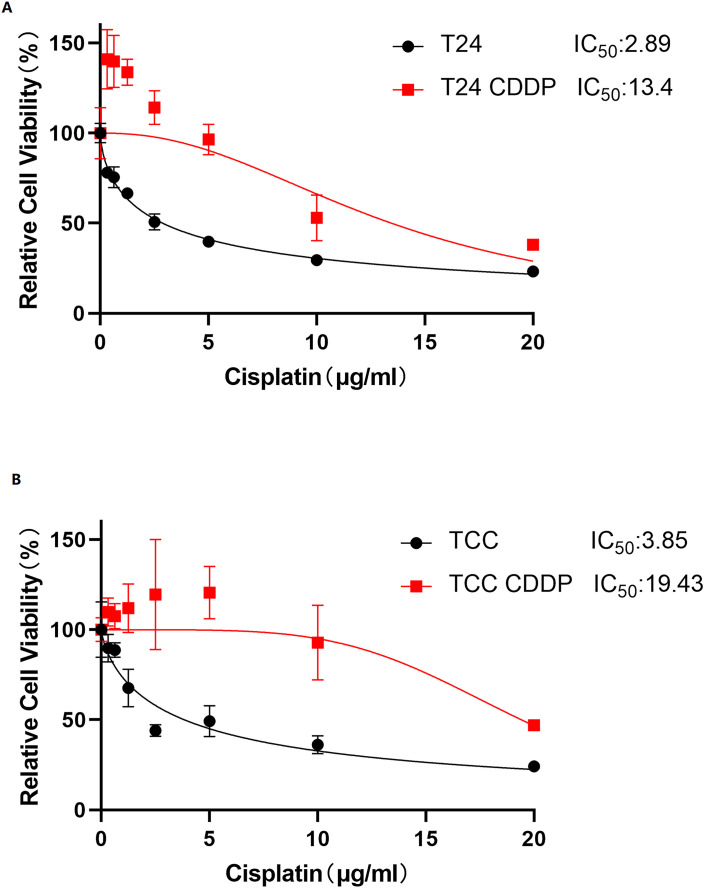
Drug Resistance of T24/CDDP and TCC/CDDP Cells to CDDP. A. After 48-hour treatment with different concentrations of CDDP (0,5,10,15,20 μg/mL), cell viability was detected using the CCK-8 method in T24 and T24/CDDP cells. B.After 48-hour treatment with different concentrations of CDDP (0,5,10,15,20 μg/mL), cell viability was detected using the CCK-8 method in TCC and TCC/CDDP cells. Data are presented as mean ± standard deviation (mean ± SD, n = 4). *P < 0.05, compared with their respective parental cells (T24/CDDP vs T24; TCC/CDDP vs TCC).

To verify the CDDP resistance in the constructed bladder cancer cell lines, we employed the CCK-8 assay to evaluate the viability of parent cells (T24, TCC) and their drug-resistant variants (T24/CDDP, TCC/CDDP) after 48-hour treatment with different concentrations of CDDP. The results demonstrated that while CDDP exhibited dose-dependent inhibition on the proliferation of both T24/CDDP and TCC/CDDP cells, their sensitivity to CDDP was significantly lower than that of the corresponding parent cells (Fig 1). Specifically, The IC50 value of CDDP in T24 cells was 2.888 μg/mL, increasing to 13.47 μg/mL in T24/CDDP cells yielding a chemoresistance.(Table 1). Similarly, the IC50 value in TCC cells was 3.849 μg/mL, which increased significantly to 19.43 μg/mL in TCC/CDDP cells, achieving a resistance index of 5.05 (Table 1). These data confirm the successful establishment of stable CDDP-resistant T24/CDDP and TCC/CDDP cell lines.

**Table 1:** The IC50 value of CDDP in T24, TCC, T24/CDDP and TCC/CDDP cells.

Cell Lines	IC50(μg/ml)	RI
T24	2.89	4.66
T24 CDDP	13.47	
TCC	3.85	5.05
TCC CDDP	19.43	

*IC50 is defined as the concentration of CDDP required to inhibit 50% of cell growth. RI = IC50 (resistant cells)/ IC50 (parental cells)

### 3.2 Inhibitory effects of nsPEF on proliferation of cisplatin-sensitive and resistant bladder cancer cells

**Fig 2:**
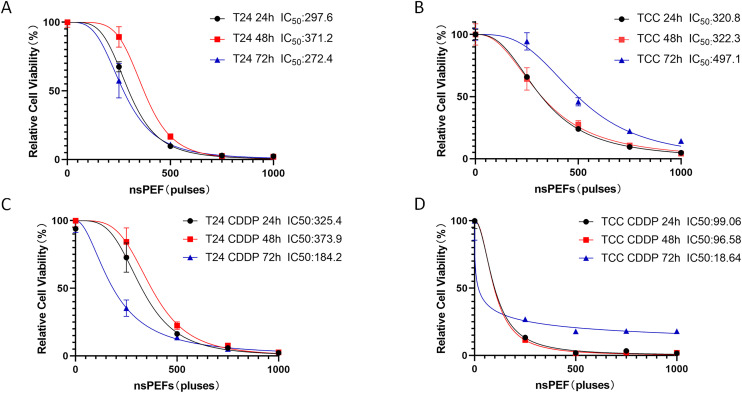
Inhibitory effects of nsPEF on the proliferation of T24 and TCC sensitive cells and their resistant counterparts. T24 and TCC sensitive cells and their resistant cells (T24/CDDP, TCC/CDDP) were treated with nsPEF at different pulse numbers (0, 250, 500, 750, 1000 pulses). Cell viability was detected using the CCK-8 method at 24, 48, and 72 hours post-treatment. Data are presented as mean ± standard deviation (mean ± SD, n = 4). P values: *P < 0.05, **P < 0.01, ***P < 0.001, ****P < 0.0001, compared to the untreated control group (0 pulses) at corresponding time points.

This study evaluated the effects of nsPEF on the proliferative capacity of cisplatin-sensitive bladder cancer cell lines (T24, TCC) and their corresponding resistant cell lines (T24/CDDP, TCC/CDDP). As shown in Fig 2, nsPEF treatment significantly inhibited the viability of both sensitive and resistant cell lines in a dose-dependent manner (increasing with pulse frequency),and the resistant cells exhibited significantly higher sensitivity to nsPEF, with more pronounced proliferation inhibition under the same treatment conditions. In general, as the nsPEF pulse frequency increased, the proliferation of both cell types gradually decreased, and the inhibitory effect on tumor cell growth became progressively stronger. Cell viability was assessed via the CCK-8 assay.

### 3.3 nsPEF promotes apoptosis in both CDDP-sensitive and drug-resistant bladder cancer cells

**Fig 3:**
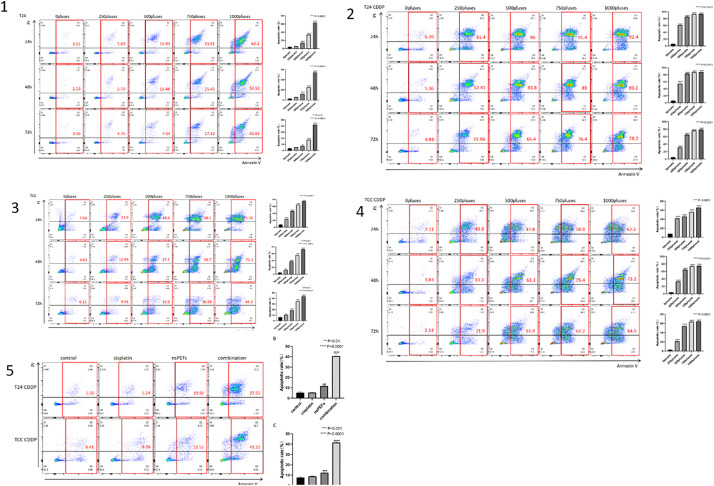
nsPEF-induced apoptosis inT24, T24/CDDP,TCC and TCC/CDDP cells T24 cells and T24/CDDP cells were treated with nsPEF at specified pulse numbers (0, 250,500,750,1000 pulses) for 24,48, or 72 hours. After treatment, the cells underwent Annexin V-FITC/PI double staining and analyzed the total apoptosis rate using flow cytometry (Q2 + Q3 quadrant, representing early and late apoptotic cells). 1 T24 nsPEF. 2 T24 CDDP nsPEF. 3 TCC CDDP nsPEF. 4 TCC nsPEF. 5 T24-TCC CDDP +CIS.

**Fig 4:**
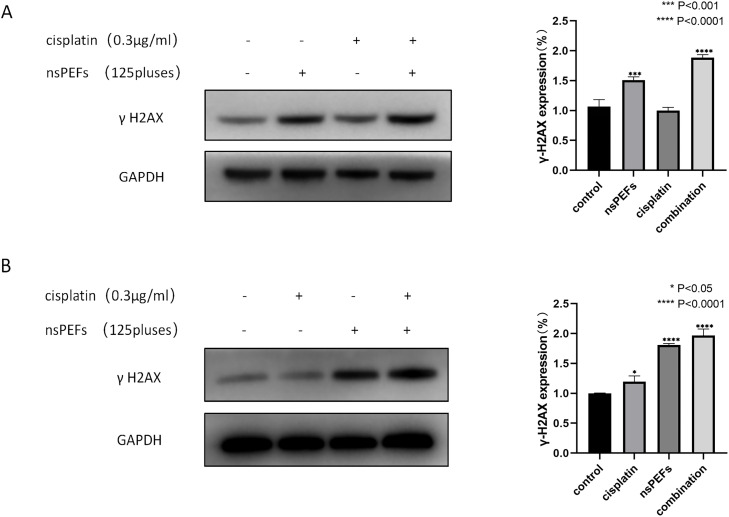
DNA Damage in T24/CDDP and TCC/CDDP Cells Induced by nsPEF. Fig 4 western blot analysis. Representative Western blot bands of γ-H2AX in the T24/CDDP treatment group (endogenous control: GAPDH). Representative Western blot bands of γ-H2AX in the TCC/CDDP treatment group (endogenous control: GAPDH). Treatment conditions: Control group (unprocessed), CDDP (0.3 μg/mL), nsPEF (125 pulse), combined group (0.3 μg/mL CDDP + 125 pulse nsPEF), treated for 48 hours.

To evaluate the effects of nsPEF on apoptosis, we treatedT24, T24/CDDP,TCC and TCC/CDDP cells with different pulse numbers (0, 250,500,750,1000 pulses) for 24,48, and 72 hours, respectively, and detected apoptosis rates using flow cytometry (Fig 3A). For T24/CDDP,TCC,TCC/CDDP cells: After 24-hour nsPEF treatment, the total apoptosis rate showed significant increases across pulse groups compared to the untreated control group (total apoptosis rate: 3.31%). Specifically, 250-pulse treatment resulted in 5.69% apoptosis, 500-pulse treatment 13.93%; 750-pulse treatment induced 33.81% apoptosis, and 1000-pulse treatment 62.1%. Although apoptosis rates maintained dose-dependent increases (higher pulse numbers led to higher apoptosis rates) at 48 and 72 hours, all pulse-treated groups exhibited significantly lower apoptosis rates than those observed at 24 hours (Fig 3). For T24/CDDP cells: nsPEF induced apoptosis trends similar to those observed in T24 cells, demonstrating both dose-dependent and time-dependent patterns. It is worth noting that under the same nsPEF processing conditions (especially with higher pulse arrays), the total apoptosis rate of Drug-Resistant cells was significantly higher than that of their parental sensitive cells (Fig 4B). We calculated the Combination Index (CI) using CompuSyn software to quantitatively confirm the synergistic effect. CI values < 1 indicate synergism, and our results show CI = 0.62 (T24 CDDP) and CI = 0.58 (TCC CDDP) for the combination of 0.3 μg/mL cisplatin and 125-pulse nsPEF, confirming significant synergistic cytotoxicity.

### 3.4 nsPEF enhanced the expression of γ-H2AX, a DNA damage marker in chemoresistant cells

To investigate the effects of nsPEF on DNA damage in drug-resistant cells, we detected γ-H2AX protein levels in T24/CDDP and TCC/CDDP cells after 48 hours using Western blotting [27,28]. The cell lines were treated with control, CDDP (0.3 μg/mL), nsPEF (125 pulse), or a combination treatment (0.3 μg/mL CDDP + 125 pulse nsPEF). As shown in Fig 4, both chemoresistant cell lines exhibited significantly higher γ-H2AX expression in the nsPEF single-drug and combined treatment groups compared to the control group, indicating that nsPEF effectively induces DNA damage in drug-resistant bladder cancer cells.

### 3.5 nsPEF Inhibits T24/CDDP Tumor Growth

**Fig 5:**
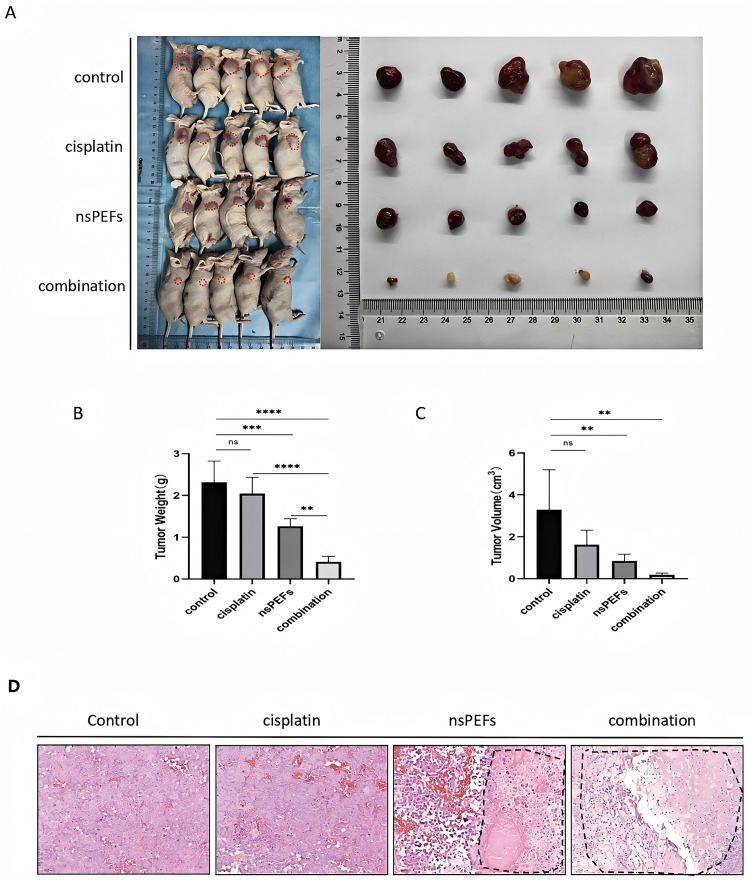
nsPEF inhibits T24/CDDP-induced tumor growth. Representative tumor images at the treatment endpoint. Tumor weights in each group (mean ± SD, n=5). Tumor volume changes in each group (mean ± SD, n=5). Hematoxylin and eosin (H&E) staining of tumor tissues (200 × magnification). Data expressed as mean ± SD. **P < 0.01, ***P < 0.001, ns: P > 0.05, compared with the control group.

To evaluate the in vivo antitumor efficacy of nsPEF, we established a nude mouse subcutaneous tumor model using T24/CDDP cells (groups: control group, single CDDP group, single nsPEF group, combined treatment group). Results showed that tumor volume and weight (Fig 5B, 5C) at the treatment endpoint (Day 14) demonstrated significant reductions in the single nsPEF group compared to the control group (3.282 ± 1.914 cm³; 2.31 ± 0.51 g) with values of 0.844 ± 0.329 cm³ and 1.26 ± 0.18 g (P < 0.01). The combined treatment group exhibited the most significant tumor suppression effects (volume: 0.181 ± 0.086 cm³; weight: 0.41 ± 0.13 g, **P < 0.001). The single CDDP group showed limited tumor suppression effects (volume: 1.623 ± 0.692 cm³; weight: 2.04 ± 0.39 g, P > 0.05). Histopathological analysis (Fig 5D) revealed that the control group exhibited deeply stained tumor cells with marked atypia and coarse granular chromatin distribution. In the single CDDP group, some cells displayed apoptotic morphology (nuclear condensation), but the overall structure remained intact. nsPEF and combined group: extensive tumor necrosis, cell nucleus fragmentation and disappearance. Safety: no obvious pathological lesions were observed in the heart, liver, spleen, lung, or kidney, indicating good safety of nsPEF combined with low-dose CDDP

### 3.6 nsPEF Regulates Transplant Tumor Proliferation, DNA Damage, and Apoptosis-Related Protein Expression

**Fig 6:**
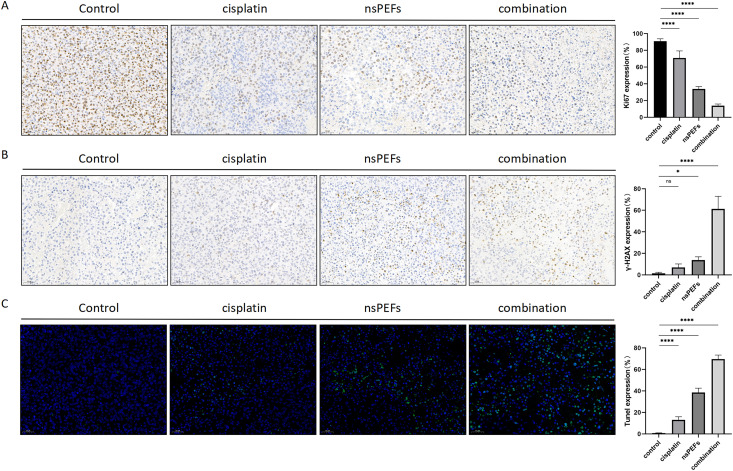
nsPEF Regulation of Key Protein Expression in Transplanted Tumors. Ki67 immunohistochemistry (brown: positive cells); γ-H2AX immunohistochemistry (brown: positive cells); TUNEL apoptosis detection (green fluorescence). Data are presented as mean ± SD. Statistical significance: ****P < 0.0001 compared to the control group.

To evaluate the antitumor efficacy of nsPEF in vivo, we conducted immunohistochemical fluorescence detection of key tumor proteins. Ki67 (proliferation) (Fig 6A): The positive rate in the control group reached 90.75 ± 2.93%. The nsPEF group (33.81 ± 2.92%) and combined treatment group (13.79 ± 2.05%) showed significantly reduced positivity rates (****P < 0.0001), indicating nsPEF’s inhibitory effect on tumor proliferation. γ-H2AX (DNA damage) (Fig 6B): The combined treatment group exhibited a significantly higher γ-H2AX-positive cell rate (61.28 ± 11.74%) compared to the control group (1.52 ± 0.91%) (****P < 0.0001), confirming that nsPEF synergizes with CDDP to exacerbate DNA damage. TUNEL (apoptosis) (Fig 6C, green fluorescence): The combined treatment group showed a TUNEL-positive cell proportion of 69.59 ± 3.80%, nearly 94-fold higher than the control group (0.74 ± 0.31%) (****P < 0.0001), suggesting that nsPEF significantly induces apoptosis and demonstrates synergistic effects with CDDP.

### 3.7 the synergistic effect of nsPEF and Cisplatin on chemoresistance cells

**Fig 7:**
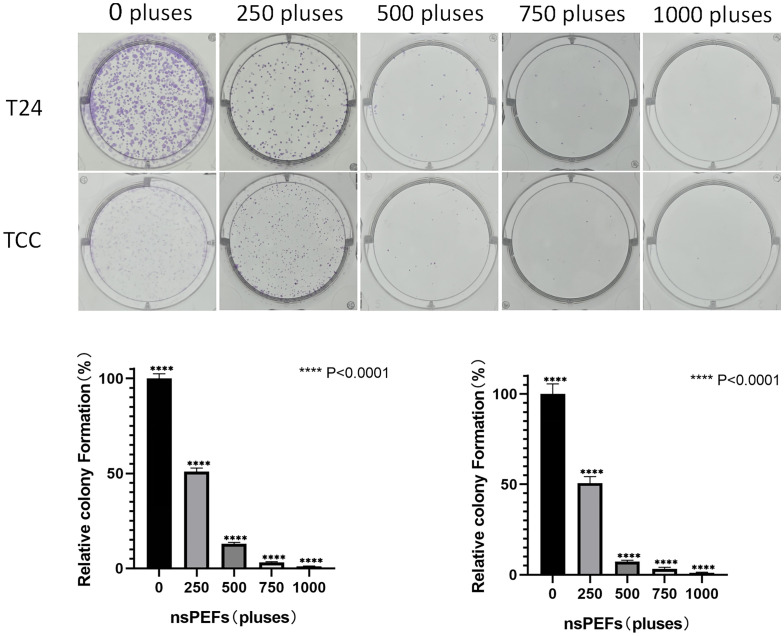
The nsPEF and cisplatin combination treatment effectively inhibits the clonogenic survival of cisplatin-resistant bladder cancer cells.

Flow cytometry (Fig 3) and clone formation data (Fig 7)demonstrated that nsPEF enhanced membrane permeabilization, which subsequently aggravated DNA damage and tumor survival, indicating the synergistic effect of nsPEF and cisplatin on chemoresistant cells

To further clarify the inhibitory effect of nsPEFs on bladder cancer, we used the clone formation assay. The results are shown in Fig 7, and T24/ TCC cells formed fewer colonies with the increasing number of electric nsPEFs, which were statistically different compared with the control group (* * * P < 0.001). Fig 7 presents the results of a colony formation assay, a critical test for evaluating the long-term proliferative potential of cancer cells after treatment.

## 4 Discussion

Bladder cancer is the tenth most common cancer in the world [1,29] and the 5-year overall survival in patients with muscle-invasive bladder cancer is very low [30]. Standard treatment for muscle-invasive bladder cancer (MIBC) is usually based on cisplatin-based chemotherapy. However, nearly half of MIBC patients develop acquired resistance [31] to the treatment of cisplatin. Cisplatin is a heavy metal complex combining divalent platinum with two chlorine atoms and two ammonia molecules, which can inhibit the replication process of DNA. It induces apoptosis [32] by acting on DNA inter-strand. The mechanisms of cisplatin resistance are multifaceted, including increased chemo efflux, increased DNA damage repair, and inhibition of the cell death pathway [33]. Therefore, platinum resistance is an important factor affecting the recurrence and survival of bladder cancer patients. Finding a new treatment or strategy to re increase the sensitivity of platinum-resistant bladder cancer patients to chemotherapy chemos will significantly improve their efficacy and increase their survival rate.

nsPEFs serve as a novel locoregional modality for cancer cell inactivation [34,35] and ablate tumor [36] by inducing cellular oxidative stress through electroporation [37], has been shown to be an effective and safe tumor ablation in animal model [38,39] and human trial [40].nsPEFs directly manipulate the cell internal [41], induce membrane perforation, and cause various cellular responses [34,42], usually with low pulse or low frequency electric field synergistic administration, strong nanosecond pulse electric field directly cause cell apoptosis or necrosis to induce cell death [43]. In addition to the perforating membrane, other mechanisms have been reported to be related to the biological effects of hundreds of kV electric fields per centimeter caused by nsPEFs, such as inhibition of voltage-gated Na+ and Ca2 + transmembrane currents [44], cellular electroformation of [45], and [46] formation of reactive oxygen species (ROS).

Combining other chemical or physical methods with nsPEFs techniques is one of the most promising approaches. Wang et al. [47] studied the combination of nsPEFs with low concentrations of gemcitabine in human oral squamous cell carcinoma. This study demonstrated that the combination therapy showed a synergistic effect on cancer cell necrosis. C.M.Edelblute et al [48] used moderate calories to enhance the efficacy of nsPEF in SCC, yielding better tumor regression efficacy and higher survival. Each of these treatments used a lower electric field intensity or less times, but achieved better results on treatment [37,38]. Moreover, nsPEFs with a certain electric field intensity can also be combined with low-dose paclitaxel [49], baicalin [50], and neutrophil membrane-coated nanoparticle [51] to improve the efficacy of tumor treatment.

T24 and TCC are relatively common bladder cancer cell lines, while the induction to establish the corresponding cisplatin resistant cells is one of the basis of this research. We follow the chemo concentration gradual induction of T24 and TCC cells. This method induced chemo resistant cell lines have higher stability and success rate [52]. To achieve the resistance index reported in the literature, the entire induction process lasted for nearly 20 months, finally establishing the corresponding cisplatin-resistant cell lines T24 CDDP and TCC CDDP. Based on the aforementioned bladder cancer cisplatin-sensitive strains and resistant cell lines, we performed subsequent experiments. To explore the role of nsPEFs for different bladder cancer, we through CCK 8 experiment, flow and clone formation experiments found that high frequency of nsPEFs can directly kill tumor cells, low frequency of nsPEFs can significantly increase the cisplatin to bladder cancer cisplatin resistant cell lines, namely in the chemo resistant cell line low concentration of cisplatin can play a killing effect, to a certain extent reversed the cell resistance. By comparing the γ -H2AX protein expression after different treatments, we showed that nsPEFs can increase DNA damage and increase DNA damage after cisplatin treatment to apoptosis. Clone forming assay proved that the nsPEF-cisplatin synergy is not merely a short-term cytotoxic effect but fundamentally compromises the reproductive integrity and self-renewal capability of the resistant cancer cell population. The ability to form colonies is a hallmark of cancer stem-like cells and treatment-resistant clones. By virtually eliminating this capacity, the combination therapy targets the root of tumor recurrence and durability of resistance. This finding strongly supports the potential of nsPEF as a sensitizing agent that can extend the therapeutic efficacy of cisplatin to otherwise resistant cells, impacting long-term disease control.

The previous experimental results [53–55] have shown that nsPEFs combined with cisplatin improves the effect of cisplatin resistance in bladder cancer, so we need to further verify at the animal experimental level. In this experiment, we used T24 CDDP by subcutaneous inoculation with cisplatin resistant cells in nude mice.

Following subcutaneous inoculation, we established tumor-bearing models in nude mice to evaluate the efficacy of nsPEFs combined with cisplatin. Tumor growth was monitored and the data revealed a marked suppression of tumor progression in the treatment group receiving both nsPEFs and cisplatin, compared to control groups receiving cisplatin alone or no treatment. This observation supports the hypothesis that nsPEFs enhance cisplatin sensitivity in resistant cells, as evidenced by reduced tumor volumes and histological analyzes showing decreased proliferation and increased apoptosis in treated tissues. Furthermore, the consistency between these in vivo findings and prior in vitro studies underscores the translational potential of this combination therapy for overcoming cisplatin resistance in bladder cancer.

The results of the above in vivo experiments are basically consistent with the results of in vitro cell experiments, namely, nsPEFs combined with cisplatin has a stronger effect on anti-resistant cells compared with the single treatment group. Comparing the morphology of all organs in nude mice before and after chemo administration, we found that there was no significant difference between the combined group and the control group, single cisplatin group and single nsPEFs group, which were normal, suggesting that the toxic and side effects of the combined group were very similar to those of other groups. Because cisplatin has certain toxicity, we found that low frequency nsPEFs combined with cisplatin can not only achieve the effect of tumor inhibition, but also reduce the toxic and side effects of chemo dose.

We investigate the possible mechanism on the capacity of ultra-short (nanosecond-scale) high-intensity electrical pulses to create electrical pores in cell membranes. By altering membrane permeability and modulating intracellular signaling pathways, it enhances organelle function while effectively inducing apoptosis in cancer cells. Our data showed the combination of cisplatin and nsPEF generates a synergistic anticancer effect. Membrane permeabilization induced by nsPEF-mediated electroporation enhances the permeability of cancer cell membranes, allowing more cisplatin molecules to penetrate cancer cells. This increases cisplatin concentration within the tumor, thereby intensifying its DNA-damaging effects. On the other hand, nsPEFs and cisplatin induce apoptosis through distinct signaling pathways. This dual-action approach overcomes drug resistance issues that might arise with single-agent therapies.

The innovative combination of nsPEF with drug delivery systems and therapeutic devices holds translational significance for clinical oncology. The non-thermal effects of nsPEF enable precise tumor targeting, significantly reducing systemic toxicity associated with high-dose chemotherapy while improving patient tolerance. NsPEF enhanced membrane permeability and resistance reversal effects of low-dose cisplatin via nsPEF reactivates tumor sensitivity, effectively overcoming limitations of single-agent therapies. This physicochemical synergy not only provides a minimally invasive treatment pathway for locally advanced or recurrent bladder cancer but also promises to shorten treatment duration and reduce recurrence risks. Future research should focus on optimizing spatiotemporal parameters for optimal pulse intensity and drug concentration, refining personalized treatment protocols, and advancing this combined therapy technology in precision oncology.

The significant synergistic cytotoxicity between nanosecond pulsed electric field (nsPEF) ablation and cisplatin (CDDP) chemotherapy, as confirmed by CI values of 0.62 (T24 cells) and 0.58 (TCC cells), highlights a promising strategy for enhancing tumor cell killing efficiency while mitigating the limitations of conventional chemotherapy. This synergism is rooted in the distinct yet complementary mechanisms of nsPEF and chemotherapeutic agents, which act on different targets to overcome tumor cell resistance and reduce drug dosage requirements. As a physical ablation energy, nsPEF exerts its effect through ultra-high voltage and ultra-short pulses, which directly disrupt the cell membrane—a critical physical barrier for maintaining cellular integrity. This membrane disruption creates transient pores or irreversible damage, altering the transmembrane potential and permeability. Unlike chemical drugs that rely on intracellular signaling pathways or metabolic processes, nsPEF’s physical attack targets the structural stability of the cell membrane, a vulnerability that cancer cells cannot easily evade even when they develop resistance to chemotherapeutics. Notably, cancer cells often acquire resistance to chemotherapeutic agents like CDDP by reducing intracellular drug accumulation, enhancing DNA repair mechanisms, or upregulating drug efflux pumps. However, these resistance mechanisms are ineffective against the physical membrane damage induced by nsPEF. Instead, the membrane disruption caused by nsPEF facilitates the intracellular entry and nuclear translocation of CDDP, overcoming the drug’s limited permeability and enhancing its cumulative toxic effects on cancer cells. This physical-chemical synergy allows for a significant reduction in CDDP dosage while maintaining or improving tumor-killing efficacy. The reduced CDDP dosage offers dual clinical benefits: first, it minimizes the severe systemic toxic side effects associated with high-dose chemotherapy, such as nephrotoxicity, neurotoxicity, and myelosuppression, thereby improving patient tolerance and quality of life. Second, lower drug concentrations reduce the selective pressure for the development of drug-resistant tumor cell clones, prolonging the therapeutic window of CDDP and preventing disease recurrence. The optimal 125-pulse nsPEF parameter identified in this study further supports the translatability of this combination strategy, as it balances efficacy and safety by maximizing synergism without excessive physical damage to surrounding normal tissues. The synergistic effect observed in both T24 and TCC cells, with a slightly more pronounced effect in TCC cells (lower CI value), suggests that this combination strategy may be applicable to multiple bladder cancer subtypes. This versatility stems from the universal vulnerability of cancer cell membranes to nsPEF-induced damage, regardless of their genetic background or chemoresistance status. By targeting distinct biological processes—physical membrane disruption by nsPEF and DNA damage by CDDP—the combination achieves a higher tumor cell killing efficiency through a “dual-target” mechanism that circumvents single-agent limitations.

In summary, this study investigates the cytotoxic effects of nsPEF on UBC cells, evaluates its inhibitory efficacy against drug-resistant UBC. Preliminary experiments revealed that nsPEF effectively suppresses bladder cancer cell proliferation. Notably, the combination of nsPEF with low-dose CDDP demonstrated significant synergistic cytotoxicity in drug-resistant UBC cells, providing novel therapeutic approaches for patients intolerant to or resistant to CDDP. Additionally, both single treatment with nsPEF and combined treatment with nsPEF and CDDP significantly increased the expression of DNA damage marker protein γ-H2AX, suggesting that enhanced DNA damage may constitute a key mechanism by which nsPEF overcomes bladder cancer resistance.

### Limit and future work

The current research focused on investigating nsPEF caused DNA damage and apoptosis. The data demonstrate that nsPEF enhances cisplatin-induced apoptosis in resistant cells, which is supported by elevated TUNEL-positive cells and γ-H2AX expression. In the future we will explore multiple cell death pathways, including ferroptosis and autophagy. Future studies should focus on optimizing nsPEF parameters for different tumor types and validating this combination strategy in in vivo models to pave the way for clinical translation.

## Conclusion

(1) The combination of cisplatin and nsPEFs exerts a synergistic anticancer effect.
(2) nsPEFs enhances the killing effect of cisplatin in chemo-resistant strains of bladder cancer, and can act as a cisplatin sensitizer, providing potential therapeutic options for enhancing the anti-antitumor effect of cisplatin and other chemotherapy chemos and reducing side effects in the treatment of bladder cancer.
(3) nsPEF enhances membrane permeability and induces apoptosis, allowing more cisplatin molecules to penetrate chemoresistant cancer cells. This increases cisplatin concentration within the tumor, thereby intensifying its DNA-damaging effects. On the other hand, nsPEFs and cisplatin induce apoptosis through distinct signaling pathways. This dual-action approach overcomes drug resistance issues that might arise with single-agent therapies.

This study demonstrates that the combination of nsPEF and CDDP exerts significant synergistic cytotoxicity against bladder cancer cells by leveraging the physical membrane-disrupting capacity of nsPEF to enhance CDDP’s intracellular efficacy. This strategy not only improves tumor-killing efficiency but also enables dose reduction of chemotherapeutics, addressing the key challenges of drug resistance and toxic side effects in cancer treatment.
